# Prions *Ex Vivo*: What Cell Culture Models Tell Us about Infectious Proteins

**DOI:** 10.1155/2013/704546

**Published:** 2013-10-26

**Authors:** Sybille Krauss, Ina Vorberg

**Affiliations:** ^1^Deutsches Zentrum für Neurodegenerative Erkrankungen e.V., Sigmund-Freud-Street 25, 53127 Bonn, Germany; ^2^Deutsches Zentrum für Neurodegenerative Erkrankungen e.V., Ludwig-Erhard-Allee 2, 53175 Bonn, Germany; ^3^Department of Neurology, Rheinische Friedrich-Wilhelms-Universität Bonn, Germany

## Abstract

Prions are unconventional infectious agents that are composed of misfolded aggregated prion protein. Prions replicate their conformation by template-assisted conversion of the endogenous prion protein PrP. Templated conversion of soluble proteins into protein aggregates is also a hallmark of other neurodegenerative diseases. Alzheimer's disease or Parkinson's disease are not considered infectious diseases, although aggregate pathology appears to progress in a stereotypical fashion reminiscent of the spreading behavior ofmammalian prions. While basic principles of prion formation have been studied extensively, it is still unclear what exactly drives PrP molecules into an infectious, self-templating conformation. In this review, we discuss crucial steps in the life cycle of prions that have been revealed in *ex vivo* models. Importantly, the persistent propagation of prions in mitotically active cells argues that cellular processes are in place that not only allow recruitment of cellular PrP into growing prion aggregates but also enable the multiplication of infectious seeds that are transmitted to daughter cells. Comparison of prions with other protein aggregates demonstrates that not all the characteristics of prions are equally shared by prion-like aggregates. Future experiments may reveal to which extent aggregation-prone proteins associated with other neurodegenerative diseases can copy the replication strategies of prions.

## 1. Prions—Infectious Agents Composed Predominately of Protein

Prion diseases or transmissible spongiform encephalopathies (TSEs) are invariably fatal neurodegenerative diseases that are associated with severe spongiform vacuolation and nerve cell loss [1]. Animal and human TSEs are infectious diseases that either naturally spread between individuals of the same species or have been accidentally transmitted through food contaminants, blood and medical products, or during surgery [1]. Animal prion diseases include scrapie in sheep and goats, bovine spongiform encephalopathy, and chronic wasting disease in elk and deer [1]. TSEs in humans also occur sporadically or are of familial origin. Human prion diseases manifest as Creutzfeldt-Jakob disease, fatal familial insomnia, Gerstmann-Sträussler-Scheinker syndrome and Kuru. Familial prion diseases are associated with dominantly inherited mutations in the coding region of the cellular prion protein. Many prion strains have also been successfully adapted to laboratory animals. Prion strains can be propagated in the same inbred mouse lines, where they produce phenotypically distinct neurological diseases [2]. Interestingly, prion strains selectively target specific brain regions, while leaving others unaffected [3, 4]. During the course of the disease, the cellular prion protein PrP^C^ misfolds and aggregates in the affected brain areas, leading to accumulation of abnormal PrP (termed PrP^Sc^) intracellularly or extracellularly. PrP^Sc^ purified from brains of diseased animals is closely associated with prion infectivity, arguing for a causal relation between the conformational state of the protein and its infectious properties [5]. According to the prion hypothesis [5] and compelling new evidence [6–8], prions constitute a class of proteinaceous infectious agents composed almost exclusively of protein without coding nucleic acid. For propagation, PrP^Sc^ acts as a template that catalyzes the conformational conversion of the cellular prion protein PrP^C^. The existence of prion strains that can propagate in the same inbred mouse lines has posed a conundrum to the prion hypothesis. Accumulating evidence now argues that prion strains are encoded by different PrP conformers capable of faithfully replicating their specific structure in the affected hosts. The strain-specific biological and biochemical signatures are likely enciphered by conformational variants of PrP^Sc^ [9–11]. 

The precursor of PrP^Sc^, PrP^C^, is a natively folded protein, with an unstructured amino terminal domain. Mature PrP^C^ is a glycosylphosphatidylinositol- (GPI-) anchored membrane protein that is abundantly expressed in the central nervous system but also in other tissues. The exact function of PrP^C^ has not been elucidated, but several studies suggest it has a cytoprotective function in neurons [12]. PrP^Sc^ fundamentally differs from PrP^C^ in its biophysical properties. PrP^Sc^ has a high *β*-sheet content, is insoluble in nonionic detergents, and its globular domain (approximately amino acid residues 89–230) is resistant to proteinase K (PK) [13]. Treatment of histological specimen or tissue lysates with PK is used routinely to identify prions in biological samples. It is important to note, however, that the exact PrP conformer with infectious properties has not been defined. Recent data argue that infectious PrP propagated *in vivo* and in cell culture can also be PK-sensitive, adding another layer of complexity to the characterization of infectious proteins [10, 14]. For simplicity, we will refer to infectious PrP molecules as PrP^Sc^. 

Propagation of prions is thought to occur through a process of nucleation-dependent polymerization, in which a seed of aggregated PrP^Sc^ templates the conformational conversion of its soluble homotypic isoform. The initial step of seed or oligomer formation is a slow and rate limiting process. In a subsequent elongation step, monomeric protein is recruited into the structurally ordered *β*-pleated fibrils, so-called amyloid. In a third step, secondary nucleation events such as filament fragmentation produce additional seeds that elongate and multiply [15]. Flow field-flow fractionation has recently been used to separate prions by size, demonstrating that particles composed of 14–28 monomers exhibit the highest infection properties *in vivo* [16]. Two lines of evidence argue that aggregate shearing is crucial for prion multiplication. First, aggregate fragmentation is an essential step in the so-called protein misfolding cyclic amplification (PMCA) developed by Saborio and colleagues [17]. In this assay, PrP^Sc^ present in brain homogenate serves as a template that is mixed with substrate PrP^C^ present in normal, uninfected brain homogenate. Consecutive steps of incubation and sonication catalyze PrP^Sc^ growth and segregation, leading to an exponential increase of prion polymers with infectious properties. Second, protein aggregate fragmentation is crucial for the propagation of prion-like protein aggregates in yeast and filamentous fungi [18, 19]. Prions in lower eukaryotes are protein-only epigenetic elements of inheritance that replicate by a seeded polymerization/fragmentation process similar to mammalian prions. Interestingly, yeast prions are fragmented by chaperone Hsp104 in conjunction with additional chaperones [20, 21]. Hsp104 has no homologue in mammalian cells, and the *in vivo* mechanism of mammalian prion fragmentation is so far unknown. 

## 2. PrP^Sc^ Formation in Cell Culture

The establishment of prion cell culture models has greatly enhanced our understanding of the cellular mechanisms of prion formation. However, even decades after the first successful prion infection *ex vivo*, many aspects of prion replication still remain elusive. The most puzzling observation is that the susceptibility of a given cell line can only be determined empirically. Most PrP^C^ expressing cell lines are refractory to mammalian prion infection for unknown reasons [22, 23]. Prion strains also demonstrate an exquisite host cell tropism not only *in vivo* [3, 4] but also in tissue culture cells [24–29]. Prion propagation *ex vivo* is not restricted to neuronal cells, and also epithelial cells or fibroblast cell lines are permissive to certain strains. Many prion strains from various origins have never been successfully propagated in cell culture [30]. These observations suggest that so far unidentified strain and host cell specific factors control the replication of prion strains. Because of the usually low infection rates, most studies have been performed with previously established cell lines persistently infected with prion strains RML or 22L. In the following sections, we will briefly review basic findings on the uptake, the initiation of an infection, and the propagation of prions in permanent cell culture models.

Uptake of PrP^Sc^ is an early step of prion infection and is independent of PrP^C^ expression [31]. PrP^Sc^ uptake is also observed in nonpermissive cell lines and thus not indicative of a productive infection [31, 32]. PrP^Sc^ formation in cell culture is initiated once PrP^C^ has been translocated to the cell surface [33]. Substantial amounts of newly formed PrP^Sc^ are already detectable within minutes to hours post exposure [34, 35]. Importantly, transient PrP^Sc^ formation has been demonstrated in resistant cell lines, arguing that initial seeding of endogenous PrP^Sc^ does not necessarily lead to a successful prion infection *ex vivo* [34]. The continuous presence of PrP^Sc^ over multiple cell passages is indicative of a productive infection of the cell culture. Abnormal prion protein accumulation is routinely detected by western blot analysis of cell lysates treated with PK (50 *μ*g/mL, 37°C, 1 hr) or by indirect immunofluorescence in fixed, prion infected cells following antigen retrieval by harsh denaturants such as guanidinium hydrochloride [36], formic acid [35] or partial proteolysis by proteinase K [36, 37]. In cultured cells, PrP^Sc^ is mainly confined to vesicles of the endocytic pathway, including early endosomes, recycling endosomes, and lysosomes [37–40]. Lipid rafts [41–43] and/or endocytic recycling compartments [40, 44] likely constitute sites of PrP^Sc^ formation. PrP^Sc^ produced in cell cultures has a half-life time of approximately 30 hrs [45]. Both lysosomes and autophagosomes have been implicated in PrP^Sc^ clearance [46–48]. Importantly, with one exception, prions in permanent cell lines do not induce visible morphological or pathological changes [49]. 

## 3. Sustained Propagation of Mammalian Prions in Culture

**Figure 1:**
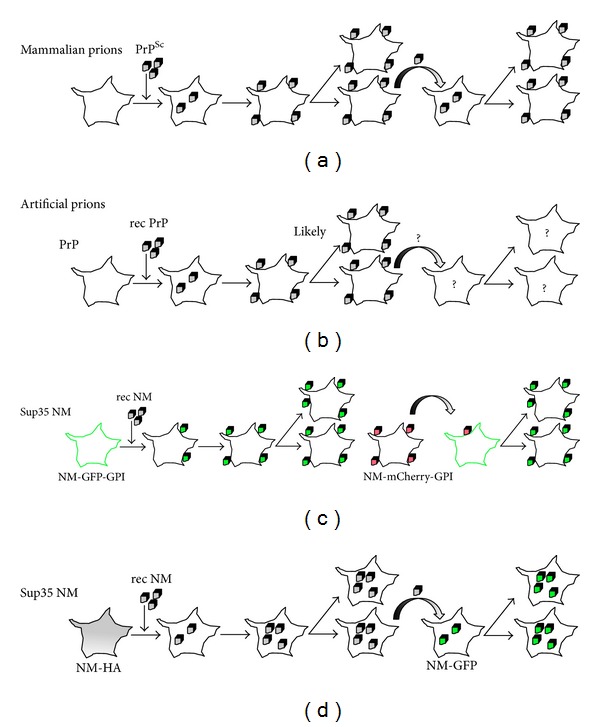
Induction of a PrP or Sup35 NM prion phenotype in mammalian cells. (a) Mammalian prions can infect selected cell cultures. Exposure of permissive cells to brain homogenate containing PrP^Sc^ leads to infection of cells. Prions persist mainly through stable inheritance of PrP^Sc^ aggregates by daughter cells. Prions also spread to adjacent cells by cell-to-cell contact or exosomes and induce productive infection in recipient cells. (b) Artificial prions produced *in vitro* from recombinant prion proteins. PrP aggregates derived from PrP and minimal components by PMCA are infectious to some cell lines. The increase in PrP^Sc^ over time suggests that prions propagate, likely by vertical transmission to daughter cells. Spreading to adjacent cells has not been studied so far. (c) Recombinant NM fibrils produce a heritable NM aggregation phenotype in N2a cells expressing a GPI-anchored NM-GFP fusion protein. When N2a cells that spontaneously form mCherry-tagged NM-GPI aggregates are cocultured with NM-GFP-GPI expressing cells, they induce NM aggregation in neighboring cells. (d) Cytosolically expressed NM-HA is soluble but can be induced to aggregate upon addition of recombinant NM fibrils. The NM aggregate phenotype is transmitted vertically and horizontally.

**Figure 2:**
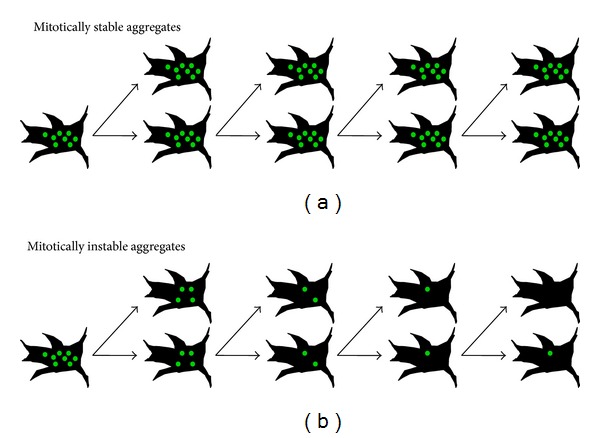
Mitotic stability of aggregate phenotypes is indicative of aggregate fragmentation. Dividing cells are a good model system to study propagation propensities of protein aggregates. (a) Proteins that can propagate as prions are mitotically stable in tissue culture, meaning that they are bidirectionally transmitted to daughter cells. Generation of infectious seeds that can self-propagate must be at least as fast as cell division. Prion aggregates must also be capable of escaping effective cellular clearance mechanisms. PrP^Sc^, NM derived prions [57, 58], and some SOD1 mutants [59] fulfill these criteria. (b) Many protein aggregates are mitotically instable. Unidirectional segregation during cell division might represent an evolutionary conserved mechanism to protect a subset of the progeny cells from toxic effects of protein aggregates. Relatively poor mitotic stability has been reported for polyQ aggregates [60, 61].

Vertical spreading from mother to daughter cells is a prominent feature of mammalian prions in tissue culture (Figure 1(a)) [50]. Under the right cell culture conditions, mammalian prions can be propagated *ex vivo* indefinitely. The mouse neuroblastoma cell line N2a infected with RML/Chandler strain in the late 1980's [51, 52] has been distributed throughout the world and still serves as the prototype cell line for studying cellular aspects of prion propagation. Cell division affects the aggregate load of the cell, diluting the number of infectious particles by half (Figure 2). The continuous prion propagation in cell culture implies that proper fragmentation and partitioning mechanisms are in place for seed multiplication. It is possible that large PrP^Sc^ aggregates are segregated by mechanical force, for example, during endocytosis, thereby producing smaller prion entities. Alternatively, unidentified cofactors catalyze the fragmentation of larger prion aggregates. Prion propagation in mammals is confined to the cell surface or endocytic vesicles, suggesting that cofactors necessary for aggregate fragmentation reside in the same cellular compartments. It is tempting to speculate that cellular quality control mechanisms are also exploited by mammalian prions to disassemble high molecular weight prion aggregates into smaller infectious entities. However, the disaggregase Hsp104 necessary for production of infectious prion entities in yeast does not exist in mammalian cells. Several chaperones have been identified that interact with or regulate PrP folding and misfolding in mammalian cells [53–55], but their contribution to prion propagation is unclear. Incomplete clearance of prion particles, for example, via lysosomes or autophagy, could potentially contribute to prion particle fragmentation. This hypothesis is supported by the observation that infection of autophagy-deficient mouse embryonic fibroblasts with mammalian prions was significantly enhanced by ectopic expression of autophagy-related protein ATG5 [56]. 

## 4. Prion Infection Spreads to Adjacent Cells

Prions *ex vivo* are not only transmitted to progeny cells, but they also spread to neighboring cells (Figure 1(a)). Dissemination of mammalian prions *in vitro* involves at least two independent routes. Horizontal transmission of prions induces a prion phenotype in the recipient cells that spreads again both vertically and horizontally. In some cell culture models, prions were secreted into the cell culture supernatant [29, 49, 63]. Several studies demonstrated that prions are often associated with exosomes released by the donor cells. Exosomes containing PrP^Sc^ have been shown to efficiently initiate prion propagation in recipient cells [64–68]. How exosomes make contact with the recipient cells and how incorporated prions then induce infection is currently unknown. Direct proximity between donor and recipient cells drastically increased the infection in other cell culture models [69]. In some instances, prions travel through cytoplasmic bridges, so-called tunneling nanotubes that form transiently between cells [70]. These data suggest that prions can utilize several distinct routes for efficient cellular spreading. So far, it is unclear if the observed differences in spreading mechanisms are due to different cell culture models or strain-specific dissemination strategies. Of note, horizontal transmission of prions in cell culture is generally much less efficient than vertical transmission to daughter cells [50].

## 5. Not All PrP Aggregates Are Infectious

The conformational transition of cellular prion protein to a misfolded, aggregated isoform is believed to be the underlying principle of prion formation, but PrP expression levels, mutations, impairment of the cellular quality control mechanisms, and some chemicals also trigger formation of PrP aggregates in cell culture. PrP^C^ expressed in cell culture is usually soluble, but overexpression increases the amount of insoluble protein (unpublished results). Prion proteins harboring familial mutations expressed in cell culture are often more abundant in detergent insoluble fractions and exhibit slightly enhanced PK resistance [71–76]. Some mutant PrP molecules linked to inherited prion diseases are retained in the cytosol, where they can aggregate into detergent-insoluble, partially PK-resistant assemblies following proteasome impairment [77]. Importantly, the moderate increase in PK resistance of PrP mutants expressed in tissue culture systems has so far not translated into infectious properties. It is possible that pathogenic mutations destabilize PrP and make it more prone to aggregate, and those misfolded proteins become refolded into an infectious PrP^Sc^ isoform in a secondary event during the very long incubation time *in vivo* [71, 74]. Of note, misfolding and aggregation are not confined to PrP with familial mutations, and replacements within the PrP coding sequence can alter PrP processing and increase PrP protease-resistance [78]. Truncated versions of PrP lacking the signal peptide and the GPI anchoring signal undergo spontaneous aggregate formation in the cytosol of mammalian cells [79]. Imbalances in cellular proteostasis can also alter the cellular localization of PrP and influence its solubility. Proteasome impairment increases the fraction of cytosolic PrP and triggers aggregation, but infectious properties of those aggregates have not been reported [80, 81]. Chemical compounds can alter the trafficking, cellular localization, and aggregation state of PrP^C^ [82–84]. The most important implication from these studies is that PrP aggregates induced in cell culture by mutations or chemicals or triggered by changes in protein homeostasis have not acquired conformations that are self-propagating. 

## 6. Recombinant Prions Induce Chronic Infections in Permissive Cell Cultures

**Figure 3:**
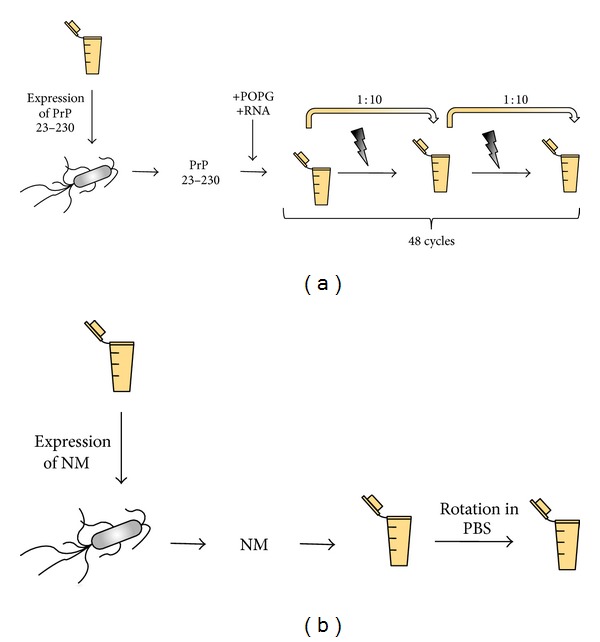
Generation of infectious protein polymers from minimal components *in vitro.* (a) For the generation of infectious PrP aggregates, purified recombinant murine PrP comprising amino acid residues 23 to 230 is mixed with lipid POPG and total liver RNA or synthetic polyA RNA. In a so-called protein misfolding cyclic amplification reaction (PMCA), intermittent pulses of sonication shear aggregates and increase the number of seeds. Preformed seeds are subsequently elongated and fragmented by serial dilution of samples into a new reaction buffer (supplemented with recombinant PrP and cofactors) and PMCA. Synthetic prions generated by this method have been shown to chronically infect SN56 and CAD cells [8, 62]. (b) The formation of Sup35 NM fibrils does not require additional cofactors. Purified recombinant NM is diluted to a concentration of 10 *μ*M in phosphate buffered saline and left at room temperature overnight. Formation of NM fibrils is expedited by agitation. Addition of these fibrils to N2a cells stably expressing soluble NM induces a heritable NM prion phenotype [57, 58].

Recently, it was shown that a synthetic prion strain, induced by the inoculation of *β*-sheet rich amyloid fibrils first into transgenic and subsequently into wild-type mice, was capable of replicating in mouse neuroblastoma cells with high fidelity [85]. New studies now demonstrate that synthetic prions derived from minimal components *in vitro* have the capacity to directly infect cell cultures (Figure 1(b)) [17, 62]. Synthetic prions produced by PMCA from recombinant mouse PrP mixed with synthetic anionic phospholipids and total liver RNA induced a sustained infection of murine SN56 cells (Figure 3(a)) [17]. Liver RNA could also be replaced by synthetic polyA RNA, leading to the formation of recombinant prions which are capable of chronically infecting a subclone of CAD cells [62]. The *ex vivo* propagation of recombinant synthetic prions produced by PMCA is surprising, given the restricted cell tropism of most prion strains. It is possible that the chosen cell lines generally represent highly susceptible substrates for prions [29, 86]. Future experiments may reveal if recombinant prions have an equally restricted host cell spectrum like natural prion strains. 

## 7. The Mammalian Cytosol Supports Propagation of Infectious Protein Aggregates

Prion diseases are the only known protein misfolding diseases that arise by aberrant folding of a GPI-anchored precursor protein. The strong association of PrP^Sc^ with membrane fractions and the attachment of PrP^C^ to the outer leaflet of the cell membrane via a GPI moiety have led to the hypothesis that GPI-anchorage of amyloidogenic proteins might be key for the infectious properties of protein aggregates. The influence of the GPI anchor on prion-like properties of aggregation-prone proteins has recently been studied by tethering the yeast prion domain of the translation termination factor Sup35 to the N2a cell membrane via a GPI anchor [87]. The fusion protein of the prion domain NM with the GPI anchor either remained soluble or spontaneously aggregated, potentially dependent on its expression level or the fused fluorescent protein ([87] and unpublished results) (Figure 1(c)). Fluorescently labeled recombinant NM fibrils prepared *in vitro* were efficiently taken up by the cells and induced aggregation of the membrane-tethered soluble NM-GFP-GPI. Once switched into the aggregated state, this conformation was faithfully inherited by daughter cells. Coculture of cells expressing spontaneously aggregating NM-mCherry-GPI with cells expressing soluble GFP-tagged NM-GPI also induced a self-perpetuating prion phenotype in the latter. 

Recent findings using the same Sup35 NM domain expressed in the cytosol of the same cell line, however, argue that cell membrane attachment via a GPI moiety is not a general requirement for infectious properties of protein aggregates in tissue culture (Figure 1(d)) [57]. In this experimental setting, the antibody epitope-tagged Sup35 NM domain was stably expressed in the cytosol as a soluble protein [79]. Spontaneous NM aggregation was not observed, not even under oxidative stress conditions [58]. Exposure of cells to *in vitro* produced fibrils from recombinant NM (Figure 3(b)) induced aggregation of the endogenous NM. Once induced, the aggregation state of NM was remarkably stable over multiple cell passages without any obvious loss of aggregate-bearing cells. NM aggregation could also be induced horizontally by direct transfer of NM aggregates from donor to acceptor through cell contact. The induced NM aggregation state in the acceptor cells was again heritable, strongly arguing that NM aggregates fulfill all criteria for prions in cell culture.

## 8. Prion-Like Properties of Proteins Associated with Neurodegenerative Diseases

Several neurodegenerative diseases are accompanied with intra- or extracellular deposition of amyloidogenic protein assemblies. While their primary sequences are diverse, aggregated proteins share a similar structure, consisting of an ordered arrangement of *β*-sheets [88–90]. Often, pathology begins locally, then progresses to other areas of the brain, reminiscent of the pathology spreading observed in prion diseases [91]. In contrast to prion diseases, neurodegenerative diseases such as Alzheimer's disease (AD), Parkinson's disease (PD), or Huntington's disease (HD) are not considered infectious diseases. Exciting research of the last years has demonstrated that aggregate pathology can spread to interconnected brain areas in several neurodegenerative disease models [91], suggesting that even nonprion protein aggregates somehow propagate and spread in tissue. These findings have led to the hypothesis that protein aggregates associated with neurodegenerative diseases share characteristic features of prions. Recent research has focused extensively on establishing cellular models for unraveling prion-like phenomena associated with neurodegenerative disease-related proteins. These studies convincingly demonstrated that aggregation-prone proteins can move between cells and seed protein aggregation in recipient cells. It is, however, a misconception that intercellular transmission and seeding of aggregates equal propagation. Propagation of prions—at least *in vitro*—is controlled by the rate of aggregate elongation, fragmentation, and degradation [15]. “Propagation” implies that transmitted seeds induce a self-templating aggregation state that will ultimately increase the number of protein aggregates per cell. Indeed, mammalian prions have found efficient ways to replicate, they produce enough seeds per cell that sustain the cellular clearance and then infect progeny and neighboring cells. This remarkable propagation efficiency is easily revealed in mitotically active cells. Here, constant cell division exponentially reduces the prion load, unless dilution and clearance effects can be compensated for by high rates of prion multiplication. The persistence of protein aggregates in dividing cells is, thus, indicative of how efficient a protein aggregate can propagate. In the following chapter we will discuss cell autonomous and noncell autonomous aggregation behaviors of the intracellular proteins Huntingtin (Htt), Tau, *α*-synuclein, and SOD1 in cell culture. We will particularly focus on the differences and similarities of PrP and other neurodegenerative disease-related proteins based on (i) their spontaneous aggregation propensities, (ii) their ability to aggregate upon addition of exogenous fibrillar seeds, (iii) their sustained propagation as induced aggregates, and (vi) their transmission in cell culture models. Of note, protein aggregates usually form in postmitotic neurons *in vivo*. However, mitotic stability *ex vivo* indicates how easily protein aggregates can propagate in a cellular environment.

## 9. Aggregation of Htt Polypeptides Encoded by Htt Exon 1

HD is a monogenic disease caused by expansion of CAG repeats in exon 1 of the Htt gene, resulting in an elongated polyglutamine (polyQ) region in the mutant protein [92]. Thus, the etiology of prion diseases crucially differs from that of HD. Mutant Htt undergoes proteolytic cleavage, giving rise to aminoterminal fragments (~the first 100–150 amino acid residues) that comprise the polyQ tract. Aggregated polyQ fragments are found in brains of HD patients and mouse models [93]. Therefore, HD pathogenesis is frequently modeled with proteins encoded by Htt exon 1. 

*(i) Aggregation Propensity When Expressed in Cell Culture.* Normal nonmutant Htt protein (polyQ proteins with 6–35 glutamine residues) is usually soluble when expressed *ex vivo*. Expression of polyQ polypeptides causes spontaneous aggregate formation in a broad variety of cell types, including nonneuronal and neuronal cells lines as well as cultures of primary neurons [92]. Aggregation kinetics, subcellular localization, and shape of the aggregates are affected by repeat lengths, presence of Htt carboxy terminal regions, expression level, expression kinetics, and cell type [94–111]. 

*(ii) Uptake and Seeding*. Similar to PrP^Sc^, recombinant polyQ peptides are internalized by cultured cells and can seed polymerization of a soluble Htt reporter protein. This has been shown by adding recombinant, fluorescently labeled polyQ fibrils to COS7, HEK293, CHO, HeLa, and N2A cells [60]. Interestingly, polyQ fibrils appeared to access the cytosol by direct penetration of the cell membrane. Internalized recombinant fibrils induced coaggregation of ectopically expressed soluble Htt fragments with nonmutant glutamine stretches. 

*(iii) Sustained Propagation*. One hallmark of PrP^Sc^ is its stable inheritance in permanent cell cultures. For Htt, the mode of inheritance by daughter cells seems less stable. Asymmetric inheritance has been suggested based on the observation that big aggresome-like structures of polyQ aggregates are transmitted to only one daughter cell [61]. Interestingly, the number of cells with induced polyQ aggregates declined exponentially upon cell division, until reaching a low, but apparently persistent steady-state level of approximately 4% above background (Figure 2(b)) [60]. 

*(iv) Cell-to-Cell Transmission*. Coculture experiments of donor HEK cells expressing GFP-HttQ71 with cells expressing soluble HttQ25 revealed very inefficient aggregate induction in recipient cells, suggesting that Htt aggregates might not be transferred between cells at high rates [60]. Cell-to-cell transmission of polyQ proteins has also been demonstrated in cocultures of human H4 glioma and HEKT cells transfected with Htt exon-1 Q103 fused to halves of the Venus fluorescent reporter by bimolecular fluorescence complementation [99]. Importantly, in this experimental setup, no direct evidence for transfer of Htt aggregates has been provided, since the transferred protein species could also be monomeric or oligomeric. To date, it has not been shown if cell-to-cell transfer of polyQ aggregates induces ongoing replication of aggregates capable of propagating vertically and horizontally like prions. 

## 10. Tau Aggregation in Cell Culture

Neurofibrillary tangles (NFTs) are a pathological hallmark of more than 20 so-called Tauopathies, including Alzheimer's disease (AD) and frontotemporal dementia. Accumulated hyperphosphorylated Tau protein aggregates into inclusions called paired helical filaments (PHF), which are the main component of NFTs. Tau is a microtubule-associated protein that stimulates and stabilizes microtubule assembly. Tau mutations cause familial neurodegenerative diseases. Tau is a naturally unfolded, highly soluble protein that exists as six isoforms [112, 113]. Alternative splicing generates Tau isoforms with three or four repeat domains (RD). The repeat domains are involved in microtubule binding and fibrillization. The mechanisms that trigger Tau conversion remain elusive. In AD brains, Tau is hyperphosphorylated, with a three- to four-fold increased phosphorylation level. Upon hyperphosphorylation, Tau dissociates from the microtubules and aggregates into the neurofibrillary tangles, resulting in microtubule destabilization and neurotoxicity [114, 115]. 

*(i) Aggregation Propensity When Expressed in Cell Culture*. Because of its high solubility, Tau—despite hyperphosphorylation—does not spontaneously aggregate upon overexpression in most cell lines [116, 117]. Spontaneous aggregation of overexpressed Tau constructs has been achieved using mutated full-length Tau and truncated Tau forms comprising the approximately 132 amino acid residue long aggregation-prone four-RD region [116, 118, 119]. 

*(ii) Uptake and Seeding*. Preformed recombinant aggregates derived from RD Tau or full-length Tau can be taken up by a variety of permanent cells and primary neurons [118, 120–122]. In primary neurons and HeLa cells, recombinant Tau was taken up by fluid-phase endocytosis [122]. Uptake of Tau appears to depend on its aggregation state, as monomeric or long fibrillar Tau is not internalized [122]. Similar to PrP^Sc^, seeding properties of internalized Tau fibrils were shown. By applying preformed recombinant full-length or Tau RD fibrils to neuronal precursor cells, HEK cells, or primary neurons, formation of intracellular Tau aggregates by recruitment of soluble Tau was induced [118, 119, 123]. Importantly, recombinant Tau fibrils could also induce endogenous mouse Tau inclusions in wild-type hippocampal neurons, arguing that seeding does not require Tau overexpression [121]. 

*(iii) Sustained Propagation*. The propagation propensity of induced Tau aggregates in cell culture has not been elucidated so far. It is unclear if the transmitted aggregates also trigger a self-catalyzed propagation of Tau aggregates. If Tau aggregates faithfully replicate upon induction like prions, they should be maintained upon continuous culture. 

*(iv) Cell-to-Cell Transmission*. Both spontaneously formed Tau RD aggregates and exogenously induced Tau full-length aggregates are transmitted from donor to acceptor cells in coculture experiments [118, 119]. Coculture of cells expressing differently tagged, spontaneously aggregating Tau RD variants revealed double stained inclusions, suggesting that Tau RD variants were exchanged between cells. This appears to depend on the release of fibrillar Tau RD species directly into the culture medium [119]. Transmission of HA-tagged Tau RD from the donor to a recipient cell line expressing both CFP- and YFP-tagged Tau RD variants increased fluorescent resonance energy transfer (FRET), suggesting that transmitted Tau RD increased endogenous Tau RD interaction and aggregate formation in the recipient. Of note, these experiments have been performed with nonphysiological, spontaneously aggregating Tau mutants, and the seeding of full-length Tau by cell-to-cell transmission has yet to be demonstrated. 

## 11. Alpha-Synuclein Aggregation in Cell Culture

Parkinson's disease (PD) is characterized by deposition of the intracellular *α*-synuclein in dense Lewy bodies or Lewy neurites. *α*-synuclein is a 14 kDa protein that plays a role in synaptic neurotransmitter vesicle trafficking and releasing [124]. Increased *α*-synuclein expression and mutations in the coding region trigger misfolding and aggregation of *α*-synuclein into oligomers and amyloid fibrils *in vivo* [125, 126]. Posttranslational modifications such as phosphorylation, oxidation, nitration, and carboxy terminal truncation have been observed *in vivo* and could affect *α*-synuclein fibrillization [127]. 

*(i) Aggregation Propensity When Expressed in Cell Culture*. Recent studies in different mammalian cell lines suggest that endogenously or ectopically expressed human *α*-synuclein exists predominately as an unfolded monomer [128]. In some cell culture models, tetrameric or oligomeric species have been identified [129–132]. Overexpression of wild-type *α*-synuclein in diverse cell lines does not generally induce inclusion body formation [133], but mutant and/or truncated *α*-synuclein forms intracellular aggregates in some but not all cellular models [134]. Most often, *α*-synuclein aggregation is induced by treatment of the cells with oxidative stress and/or increased calcium levels [135–139]. *α*-synuclein aggregation in cell culture can also be promoted by coexpression of mutant leucine-rich repeat kinase, a protein linked to familial PD [140], or by treatment with mitochondrial inhibitor rotenone [141].

*(ii) Uptake and Seeding*. *α*-synuclein seeds prepared from recombinant protein *ex vivo* can be taken up by a variety of different cells [142–146]. Endocytosis has been proposed as a possible route for internalization of fibrillar recombinant human *α*-synuclein [142, 146]. In some instances, uptake of fibrils into the cells by physiological routes failed, and fibrils had to be introduced via cationic lipid transfection [145]. Variations in uptake efficiencies might be due to differences in fibril preparation, as has been shown for different *α*-synuclein oligomers [143, 144]. Exogenous *α*-synuclein oligomers or fibrils can seed the formation of intracellular inclusions in different permanent and primary cell models [143–146]. Overexpression of wild-type or mutant *α*-synuclein [146] is not generally required for noncell autonomous aggregate induction, but aggregation kinetics appear to be faster under *α*-synuclein overexpression conditions [144, 145]. 

*(iii) Sustained Propagation*. So far, sustained propagation of induced *α*-synuclein aggregates in cell culture has not been reported. Future experiments will be necessary to evaluate if *α*-synuclein aggregates, once triggered, gain self-propagating properties that allow them to continuously propagate over multiple passages. In analogy to mammalian prions, this would require that aggregate growth and fragmentation exceed the loss of aggregates by cellular clearance or cell division. 

*(iv) Cell-to-Cell Transmission*. Coculturing experiments with cells producing *α*-synuclein aggregates with recipient cells revealed transfer of *α*-synuclein between cells with subsequent aggregate induction in the recipient cell [147, 148]. The exact mechanism of aggregate transfer has not been elucidated. Low amounts of soluble and aggregated forms of *α*-synuclein have been shown to be released by cells, potentially by unconventional endocytosis or packaged into exosomes [149–152]. 

## 12. Superoxide Dismutase 1 Aggregation in Cell Culture

Amyotrophic lateral sclerosis (ALS) is a devastating neurodegenerative disease that usually occurs sporadically but can also be of genetic origin. During the course of the disease, motor neurons in spinal cord, brain stem, and brain degenerate [153]. More than 140 mutations in the copper/zinc superoxide dismutase (SOD1), an antioxidant enzyme, are linked to approx 2% of familial ALS cases (http://alsod.iop.kcl.ac.uk/). Misfolding of mutant SOD1 and cytoplasmic inclusion of body formation underlies disease pathology [154]. 

*(i) Aggregation Propensity When Expressed in Cell Culture*. Active SOD1 is located in the cytoplasm in the form of a homodimer. Overexpression of wild-type SOD1 usually does not lead to inclusion body formation in cell culture [155]. Still, misfolded states of the wild-type or mutant SOD1 overexpressed in cell culture could be revealed by antibody detection of normally buried antibody epitopes [156]. The aggregation propensities of various SOD1 mutants differ in cell culture [59, 155, 157]. 

*(ii) Uptake and Seeding*. Aggregated recombinant SOD1 can be taken up by neuroblastoma cells [59], and primary or permanent microglia cell cultures [158]. Uptake of aggregated SOD1 was decreased by either chemical inhibition of rafts and scavenger receptors, or by macropinocytosis inhibitors, indicating that uptake mechanisms might depend on the cell line or seed preparation [59, 158]. Recombinant mutant SOD1 aggregates added to the cell culture medium gained access to the cytosol of N2a cells, where they recruited the homotypic protein into small, dispersed aggregates [59]. 

*(iii) Sustained Propagation*. Unlike polyQ aggregates, seed-induced SOD1 aggregates exhibit a remarkable mitotic stability in cell culture [59]. The stable propagation of the SOD1 aggregation phenotype over multiple passages in cell culture is reminiscent of that of mammalian prions and suggests steady state levels of SOD1 aggregates. 

*(iv) Cell-to-Cell Transmission*. Fluorescently tagged mutant SOD1 aggregates are efficiently taken up by N2a cells and subsequently transmitted to cocultured cells [59]. Direct cell contact between donor and acceptor cells was not required for transmission, suggesting that SOD1 was secreted into the cell culture medium. Secretion of endogenous SOD1 has been reported for a variety of different cell lines, including fibroblasts, neuroblastoma, motor neuron cell lines and primary spinal cord cultures [159–161]. It remains to be established if and how self-perpetuating SOD1 aggregates produced in cell culture spread horizontally and induce a SOD1 aggregation phenotype in the acceptor cells [59].

## 13. Concluding Remarks

Prions are proteinaceous infectious protein aggregates that have the capacity to enter the host cells and impose their abnormal conformational states onto their endogenous counterparts. Noncell autonomous aggregate induction through external seeds has recently also been demonstrated for a variety of different proteins associated with nonprion neurodegenerative diseases. The ability to invade cells and seed cytosolic aggregation appears to be a general feature of amyloidogenic proteins. Importantly, the aggregation state of a protein does not reflect its infectious properties and only a thorough investigation of its propagation *ex vivo* or *in vivo* can ultimately confirm infectious potentials. Prion replication proceeds through a process of seeded polymerization and secondary nucleation events by fibril fragmentation, and escape of those seeds from cellular clearance is crucial for prion maintenance. Mitotically active cells represent tractable models for studying aspects of prion fragmentation and clearance, as inefficient prion fragmentation or enhanced clearance result in rapid prion loss due to aggregate dilution by cell division. The fact that mammalian prions can successfully replicate mitotically active cells argues for a steady state between prion formation and prion reduction. The observed mitotic instability of other protein aggregates *ex vivo* could, thus, be due to inefficient fragmentation or enhanced clearance. In conclusion, the ability of a given protein aggregate to achieve a steady state of aggregate multiplication and reduction will ultimately affect its prion capacity.

*Note*. While in print persistent propagation of induced alpha-synuclein aggregates was reported in mouse neuroblastoma cell line N2a [162]. 

## Abbreviations

AD: Alzheimer's disease
ER: Endoplasmic reticulum
GdnHCl: Guanidinium hydrochloride
GPI: Glycosylphosphatidylinositol
HA: Hemagglutinin antibody epitope
HD: Huntington's disease
Htt: Huntingtin
NFT: Neurofibrillary tangles
NM: Prion domain and middle domain of the yeast Sup35 translation termination factor
PD: Parkinson's disease
PHF: Paired helical filaments
PK: Proteinase K
PMCA: Protein misfolding cyclic amplification
polyQ: Polyglutamine
PrP: Prion protein
PrP^C^: Normal cellular isoform of the prion protein
PrP^Sc^: Disease-associated isoform of the prion protein
RD: Repeat domain of Tau
TSE: Transmissible spongiform encephalopathy.
